# Association between physical exercise, executive function, and cerebellar cortex: A cross-sectional study among the elderly in Chinese communities

**DOI:** 10.3389/fnagi.2022.975329

**Published:** 2022-08-23

**Authors:** Wei Li, Yong Li, Yaopian Chen, Ling Yue, Shifu Xiao

**Affiliations:** ^1^Department of Geriatric Psychiatry, Shanghai Mental Health Center, Shanghai Jiao Tong University School of Medicine, Shanghai, China; ^2^Alzheimer’s Disease and Related Disorders Center, Shanghai Jiao Tong University, Shanghai, China; ^3^Department of Nephrology, Hubei Provincial Hospital of Traditional Chinese Medicine, Wuhan, China; ^4^Department of Nephrology, Affiliated Hospital of Hubei University of Chinese Medicine, Wuhan, China; ^5^Hubei Provincial Academy of Traditional Chinese Medicine, Wuhan, China; ^6^Department of Sleep Medicine, Wenzhou Seventh People’s Hospital, Wenzhou, China

**Keywords:** physical exercise, cognition, elderly, MRI, cerebellum cortex

## Abstract

**Background:**

Previous studies have confirmed that physical exercise may be beneficial for brain health, but there is little data on this among older Chinese.

**Objective:**

The purpose of this study was to explore the relationship between physical exercise and cognitive impairment, and to explore the possible mechanism by which physical exercise prevents cognitive decline.

**Materials and methods:**

192 older adults with dementia, 610 older adults with mild cognitive impairment (MCI), and 2,218 healthy older adults were included in the study. Through standardized questionnaires, we obtained their general demographic information (such as gender, age, education, etc.), disease-related information (hypertension and diabetes) and physical exercise information (such as whether they did physical exercise and the frequency of physical exercise, etc.). The mini-mental state examination (MMSE) and Montreal Cognitive Assessment (MoCA) were used to assess their overall cognitive function, while the Wechsler block diagram was used to assess their executive function. Moreover, 164 healthy, randomly selected older adults also underwent brain MRI scans at the same time, and the target brain regions included hippocampus, gray matter, and cerebellar cortex.

**Results:**

By using stepwise multiple logistics regression analysis, we found that physical exercise was associated with both MCI (*p* = 0.001*, *OR* = 0.689, 95%CI: 0.553–0.859) and dementia (*p* < 0.001*, *OR* = 0.501, 95%CI: 0.354–0.709), independent of gender, age, education, and other factors. The results of ROC curve showed that the area under the curve of physical exercise in predicting MCI and dementia was 0.551 (*p* < 0.001*, 95%CI: 0.525–0.577) and 0.628 (*p* = 0.001*, 95%CI: 0.585–0.671), respectively. The results of partial correlation analysis showed that physical exercise was associated with left cerebellar cortex (*r* = 0.163, *p* = 0.023), right cerebellar cortex (*r* = 0.175, *p* = 0.015) and Wechsler block diagram score (*r* = 0.235, *p* = 0.011). Moreover, the results of linear regression analysis mediation model showed that physical exercise may affect Wechsler block diagram score through influencing the thickness of right cerebellum cortex, and the latter may play a partial mediation effect (indirect *B* = 0.001, *p* = 0.045).

**Conclusion:**

Physical exercise might be a protective factor for mild cognitive impairment and dementia among the Chinese elderly, and there might be an association among physical exercise, executive function, and the thickness of the cerebellar cortex.

## Introduction

Dementia is a major public health problem that is a growing burden due to an aging society (Rossor et al., 2010). According to a meta-analysis, the prevalence of all types of dementia among people 50°years and older in communities was 697 per 10,000 people (CI95%: 546–864), and the number will roughly double every 5°years (Cao et al., 2020). Since dementia is incurable, how to prevent and delay the progression of dementia is the focus of the whole cognitive field of research (Revi, 2020). Accumulating evidence suggests that physical exercise, especially aerobic exercise, is able to produce mild-to-moderate cognitive gains in healthy adults (Smith et al., 2010). Moreover, older adults with dementia (Morris et al., 2017) and mild cognitive impairment (MCI) (Baker et al., 2010) were also observed to improve their cognitive performance after physical exercise. Therefore, the hypothesis that aerobic and intensive exercise may slow cognitive impairment in older people is widely supported. However, previous studies have tended to use only mammalian models or cell experiment [for example, Chen et al. (2022a) found that exercise can affect the complications of AD by affecting the activity of monocytes, and they also found that exercise can affect the up-regulation of miR-215 expression, thus playing a role of cognitive protection (Chen et al., 2022b)], large-scale epidemiological surveys are extremely limited and the mechanisms are also unclear (Lamb et al., 2018; Lourenco et al., 2019).

Structural magnetic resonance imaging is an important tool in cognitive research, and one of the most commonly used methods for mechanism research. Foreign observation research shows that physical activity is associated with less brain atrophy, greater brain volume, and slower dementia progression and reduced risk of dementia (Erickson et al., 2011; Morris et al., 2017). To be more specific, some studies suggest that physical exercise protects against cognitive decline by increasing hippocampal volume (Erickson et al., 2011; Yu et al., 2014), while others support that exercise may improve cognitive function by affecting the volume of the cerebellar cortex (Marques-Aleixo et al., 2016; Inoue et al., 2018). However, studies on the effects of physical exercise on brain structure are relatively rare among elderly Chinese. For example, in Tao J et al.’ study, they found that physical exercise could significantly increase gray matter volume (GMV) in the insula, putamen and medial temporal lobe (Tao et al., 2017), but sun Li et al. (2019) did not reach a similar conclusion.

In view of the rarity and inconsistency of similar studies, we carried out a cohort study specifically to investigate the effects of physical exercise on mild cognitive impairment and dementia among the elderly in the Chinese community. Meanwhile, we also conducted head MRI on some people, so as to better explore the related mechanisms of physical exercise on cognitive function, and the target brain regions mainly included hippocampus, cerebellar cortex, and gray matter. Our hypothesis was that physical exercise might be a protective factor for MCI and dementia, and the mechanism might be related to the influence of physical exercise on these cognitively related brain regions.

## Materials and methods

### Data sources

The current cross-section study was derived from the China Longitudinal Aging Study (CLAS), which has been described in detail in our previous studies (Xiao et al., 2013; Li et al., 2019). A total of 3,020 community-based individuals aged 60°years or older, including 610 patients with mild cognitive impairment (MCI), 192 patients with dementia, and 2,218 normal controls, were included in the current study. Inclusion criteria were as follows: (1) aged 60 and above; (2) without acute, unstable, or terminal physical illness; (3) without serious mental illness, such as severe depression and schizophrenia. Exclusion criteria included: (1) incomplete information; (2) were not able to sit on a chair and walk 10 feet (3.05°m) without assistance; (3) suffer from sarcopenia, physical disability or other conditions that affect physical activity in older adults; (4) participants or their guardians declined to be interviewed. Once subjects meet the criteria for enrollment, they would undergo completed physical examinations, laboratory tests, and cognitive assessments. All diagnoses were done by experienced geriatricians. Moreover, Brain MRI scans were also performed on 164 older adults with normal cognitive function in a random lottery (of the 164 elderly, 84 were males, and 80 were females. Their average age were 68.65 ± 7.324°years, their average years of education were 8.87 ± 3.79° years).

The study was approved by the Ethics Committee of Shanghai Mental Health Center, and all subjects signed informed consent before the study began. The whole study was carried out in accordance with the principles of the Declaration of Helsinki.

### Diagnostic criteria

#### Mild cognitive impairment

The diagnosis of mild cognitive impairment was based on Petersen’s criteria (Petersen, 2011): (1) the main complaint was memory loss, which could be confirmed by a family member or relative; (2) other areas of cognitive function were relatively unaffected or only slightly affected; (3) daily life was not affected; (4) did not meet the diagnostic criteria for dementia; (5) symptoms were not caused by another disease; (6) the memory test score was at least 1.5 standard deviations lower than the average score for individuals of the same age and education level.

#### Dementia

The diagnosis of dementia was in accordance with the Diagnostic and Statistical Manual of Mental Disorders, fifth edition (DSM-V) (Biedermann and Fleischhacker, 2016): (1) one or more cognitive domains (complex attention, executive ability, learning and memory, language, sensory perception, and social cognition) were significantly reduced compared to the past; (2) impaired ability to perform daily living; (3) cognitive damage was not in delirium stage; (4) the above damage could not be explained by other mental and emotional diseases (such as depression, schizophrenia, etc.).

### Cognitive assessment

The Mini-Mental State Examination (MMSE) (Folstein et al., 1975) and Montreal Cognitive Assessment (MoCA) (Nasreddine et al., 2005) were used to measure the subjects’ overall cognitive function. The above two scales are the most commonly used tools in the cognitive field and both have good sensitivity and specificity. However, compared with MMSE, MoCA scale has better sensitivity. Therefore, the former was mainly used to screen for dementia, while the latter was mainly used to screen for MCI (Ciesielska et al., 2016). Wechsler block diagram was used to assess executive function, lower scores for block diagram would indicate more severe impairment of executive function (Li et al., 2017). At the same time, we also used the Geriatric Depression Scale (GDS) to exclude depression and GDS greater than or equal to 10 was considered to have depressive symptoms (Benedetti et al., 2018).

### MR image acquisition and processing

Baseline cranial MRI was performed in 164 elderly subjects with normal cognitive function there were 84 males (51.2%), with an average age of 68.65 ± 7.32°years and an average length of education of 8.87 ± 3.79°years). T1 structural images were obtained using the Magnetom Verio 3.0T scanner (Siemens, Munich, Germany), and the parameters were as follows: *TR* = 2,300 ms, *TE* = 2.98 ms, flip angle 9°, slice thickness 1.2°mm, matrix size 240*256, field of view (FOV)240*256 mm, and 17,614 slices. All sMRI data was processed using FreeSurfer V6.0 software Clinica (Brown et al., 2020), including spatial registration, cortical thickness estimation, cortical surface segmentation, extraction of subcortical structures, and inclusion of blocks to 46 global structures. Based on previous studies, the hippocampus and cerebellum cortex were considered as our core research brain area (Cotterill, 2001; Wang et al., 2015; Tomiga et al., 2020).

### Definition of physical exercise

The definition of physical exercise in this study was according to ACSM (Garber et al., 2011): (1) Time: ≥ 20 min/day; (2) Intensity: moderate intensity (such as jogging, fast walking, stair climbing, etc.) and/or intense intensity (such as basketball, rope skipping, long-distance running, etc.); (3) Frequency: ≥ 4°days/week. Those who (1) exercised less than 20 min a day, (2) lower than moderate intensity; (3) frequency < 1°day/week; (4) with diseases that were not suitable for exercise, such as broken bones, asthma; were defined as without physical exercise (Lin et al., 2019). In the current study, the main variables we investigated included: (1) physical activity; (2) If yes, the number of years and frequency of exercise.

### Covariates

By using a standardized questionnaire, we also collected their general demographic information (age, gender, and education), lifestyle information (smoker, drinker, tea drinker, hobby: In the current study, hobbies were those brain-enhancing interests, such as reading books, playing music and using computers, that lasted at least a year) and disease-related information (hypertension and diabetes). Of these variables, those that were differences among the MCI group, the dementia group, and the normal control group were considered as covariables.

## Statistical methods

Continuous variables were expressed by Mean ± standard deviation, and classified variables were expressed by frequency (%). Univariate ANOVA and Chi-square test were used to compare continuous and categorical variables among the MCI group, dementia group, and normal control group, respectively. Stepwise multivariate logistic regression analysis was used to explore the association between physical exercise and cognitive impairment: model 1 only contained physical exercise; model 2 contained physical exercise, age, education, and gender, model 3 contained physical exercise, age, education, male, smoker, tea, hobby, diabetes, and hypertension (Stepwise multiple logistic regression analysis was chosen because age, sex, and education were recognized variables affecting cognition, while other variables were included in consideration of their possible interaction with these variables. Finally, the selection of these covariates was also based on previous research by us and others). Next, the ROC curve was used to explore the sensitivity and specificity of physical exercise in predicting MCI and dementia. Then partial correlation analysis was used to explore the correlation between physical exercise and cognitive scores and cognitive brain regions, and controlled for age, education, and gender. Finally, the mediation model of linear regression analysis was further used to explore the relationship between physical exercise, cerebellum cortex and Wechsler block diagram score (Although there were differences in brain regions other than the cerebellar cortex between exercise and non-exercise elderly people, there was no obvious correlation with cognitive scores, so we did not show this in the paper). Two-tailed tests were used at a significance level of *p* < 0.05 for all analyzes. The data was analyzed using SPSS 22.0 (IBM Corporation, Armonk, NY, United States).

## Results

### The general demographic data of the study population

Table 1 presents the differences in general demographic information among the three groups (Dementia, MCI, and Normal). The proportion of physical exercise was significantly higher in the normal elderly (76.1%) than in the dementia (47.9%), and MCI (63.8%) groups. Moreover, there were also statistical differences in age (*p* < 0.001), education (*p* < 0.001), gender (*p* < 0.001), smoker (*p* = 0.009), tea drinker (*p* < 0.001), hobby (*p* < 0.001), diabetes (*p* < 0.001), hypertension (*p* = 0.006), MMSE (*p* < 0.001), and MoCA (*p* < 0.001) among the three groups.

**TABLE 1:** Demography, life style, physical diseases, and cognitive function in the overall database of study participants.

Variables	MCI (*n* = 610)	Dementia (*n* = 192)	Normal (*n* = 2218)	F/X^2^	*p*
Age, years	73.86 ± 8.24	78.83 ± 7.54	70.10 ± 7.53	145.76	< 0.001*
Education, years	5.67 ± 5.02	4.34 ± 4.77	9.25 ± 5.72	138.34	< 0.001*
Males, n (%)	233 (38.2)	71 (37.0)	1074 (48.4)	26.347	< 0.001*
Smoker, n (%)	147 (24.1)	43 (22.4)	650 (29.3)	9.46	0.009*
Drinker, n (%)	119 (19.5)	31 (16.1)	475 (21.4)	3.65	0.162
Tea drinker, n (%)	220 (36.1)	57 (29.7)	1116 (50.3)	61.39	< 0.001*
Hobby, n (%)	267 (43.8)	48 (25.0)	1318 (59.4)	116.98	< 0.001*
Diabetes, n (%)	108 (17.7)	49 (25.5)	346 (15.6)	13.136	< 0.001*
Hypertension, n (%)	294 (48.2)	112 (58.3)	1029 (46.4)	10.244	0.006*
Physical exercise, n (%)	389 (63.8)	92 (47.9)	1689 (76.1)	94.35	< 0.001*
MMSE	22.38 ± 5.73	13.97 ± 7.41	26.80 ± 3.51	877.04	< 0.001*
MoCA	16.72 ± 6.15	9.10 ± 6.25	22.79 ± 5.18	724.15	< 0.001*

*Means *p* < 0.05.

MoCA, Montreal Cognitive Assessment; MMSE, Mini-mental State Examination; MCI, mild cognitive impairment.

### Possible factors associated with mild cognitive impairment and dementia by stepwise multivariate logistic regression models

**FIGURE 1:**
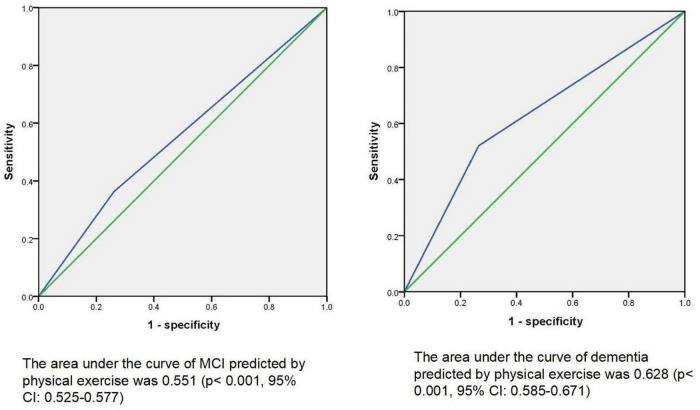
ROC curve of physical exercise predicting cognitive impairment.

Stepwise multivariate logistic regression models were used to explore the possible factors that might be associated with MCI and dementia. In model 1, without controlling for any variables, we found that physical activity was an influential factor for both MCI (*p* < 0.001* *OR* = 0.551, 95%CI: 0.455–0.668) and dementia (*p* < 0.001*, *OR* = 0.288, 95%CI: 0.214–0.389); In model 2, after controlling for age, education, and gender, we found that physical activity was also an influential factor for both MCI (*p* < 0.001*, *OR* = 0.670, 95%CI: 0.540–0.830) and dementia (*p* < 0.001*, *OR* = 0.442, 95%CI: 0.315–0.621); In model 3, smoker, tea drinker, hobbies, diabetes, and hypertension were further controlled on the basis of Model 2, and the above relationship still existed (MCI: *p* = 0.001*, *OR* = 0.689, 95%CI: 0.553–0.859; dementia: *p* < 0.001*, *OR* = 0.501, 95%CI: 0.354–0.709). Table 2 presents the results. Then ROC curve was used to investigate the sensitivity and specificity of physical exercise in predicting MCI and dementia, and the area under the curve was 0.551 (*p* < 0.001*, 95%CI: 0.525–0.577) and 0.628 (*p* = 0.001*, 95%CI: 0.585–0.671), respectively. Figure 1 presents the results. However, we did not find the effect of frequency and duration of physical exercise on cognitive impairment.

**TABLE 2:** Physical exercise (as categorical variable) and mild cognitive impairment/dementia.

Physical exercise	*B*	S.E	Wald	df	*p*	*OR*	95% confidence interval
**MCI**							
Model 1	−0.595	0.098	37.032	1	< 0.001*	0.551	0.455–0.668
Model 2	−0.401	0.110	13.333	1	< 0.001*	0.670	0.540–0.830
Model 3	−0.372	0.112	11.027	1	0.001*	0.689	0.553–0.859
**Dementia**							
Model 1	−1.244	0.153	66.300	1	< 0.001*	0.288	0.214–0.389
Model 2	−0.815	0.173	22.260	1	< 0.001*	0.442	0.315–0.621
Model 3	−0.691	0.177	15.202	1	< 0.001*	0.501	0.354–0.709

*Means *p* < 0.05.

MCI, mild cognitive impairment; model 1 only contained physical exercise, model 2 contained age, education, male and physical exercise, model 3 contained age, education, male, smoker, tea, hobby, diabetes, hypertension, and physical exercise.

### The connection between physical exercise and brain structure

**FIGURE 2:**
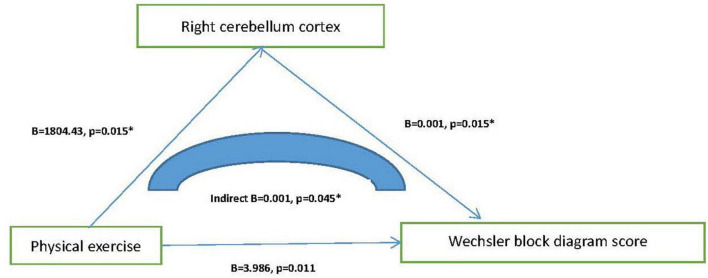
Mediating effect model among physical exercise, cerebellum cortex, and Wechsler block diagram score.

To explain the possible mechanisms by which physical exercise affects cognition, we added structural magnetic resonance data. By using correlation analysis, we found that physical exercise was positively correlated with left cerebellum cortex (*r* = 0.163, *p* = 0.023), right cerebellum cortex (*r* = 0.175, *p* = 0.015), and Wechsler block diagram score (*r* = 0.235, *p* = 0.011). Table 3 presents the results. Then the mediation model of linear regression analysis was further used to explore the relationship between physical exercises, cerebellum cortex and Wechsler block diagram score, we finally found that physical exercise may affect Wechsler block diagram score through influencing the volume of right cerebellum cortex, and the latter may play a partial mediation effect (indirect *B* = 0.001, *p* = 0.045). Figure 2 presents the results.

**TABLE 3:** Correlation between physical exercise and brain structure and cognitive scores.

Variables	Variables	*r*	*p*
Physical exercise	Left hippocampus	0.810	0.260
	Right hippocampus	0.080	0.268
	Left cerebellum cortex	0.163	0.023*
	Right cerebellum cortex	0.175	0.015*
	Total gray	0.054	0.400
	MMSE	0.137	0.138
	MoCA	0.129	0.164
	Wechsler block diagram	0.235	0.011*

*Means *p* < 0.05.

MoCA, Montreal Cognitive Assessment; MMSE, Mini-mental State Examination; MCI, mild cognitive impairment.

## Discussion

Given the evidence that physical activity is associated with improvements in all aspects of health, interventions that explore ways to reduce the risk of developing dementia and improve outcomes in people with dementia are critical (Ahlskog et al., 2011). In the current study, we investigated the association between physical activity and cognitive impairment among the elderly in the Chinese community and found that (1) physical activity was a common contributor to both MCI and dementia and was likely to have a positive effect; (2) physical exercise mainly improved executive function, and the volume of right cerebellar cortex probably played a part in mediating this process.

In our current large cross-sectional cohort study, we explored the association between physical exercise and cognitive impairment among older adults in the community (including 192 older adults with dementia, 610 older adults with MCI, and 2,218 normal elderly adults). We found that people with mild cognitive impairment or dementia had significantly lower rates of physical exercise than normal controls. After controlling for age, sex, education, and diseases, physical exercise remained an important predictor of MCI and dementia. In addition, the results of the ROC curve also showed that physical exercise had moderate predictive power for MCI or dementia. However, we did not find an association between the duration or frequency of exercise and cognitive function. In Lin sun’ study, they found that physical exercise has a beneficial effect on cognition, particularly visuospatial function, and decreases the risk of dementia in a Non-dementia Aging Chinese Population (Lin et al., 2019). In Han et al.’s (2021) study, they found that physical exercise had an intermediate effect on cognitive status among the elderly in the Chinese community. Therefore, our findings were consistent.

In order to further explore the possible mechanism of physical exercise preventing cognitive impairment, 164 older adults with normal cognitive function underwent brain MRI of T1 phase, and the target brain regions mainly included hippocampus, cerebellar cortex, and gray matter. We finally found that physical exercise was positively correlated with left cerebellum cortex, right cerebellum cortex, and Wechsler block diagram score, but not with hippocampus volume, gray matter volume, MMSE, and MOCA. Next, we further explored the internal relationship between physical exercise, cerebellar cortex, and Wechsler block diagram. Through the mediation analysis of linear regression, we finally found that physical exercise could effectively affect Wechsler block diagram score (executive function), and in this process, the right cerebellar cortex was likely to play a mediating effect. However, we did not find that the left cerebellar cortex played an important role in this process.

Although the cerebral cortex has been extensively studied, little is known about the cerebellar cortex (Van Essen et al., 2018). The cerebellar cortex is subdivided mediolaterally and rostrocaudally into a reproducible array of zones and stripes, which makes the cerebellum a valuable model for studying pattern formation in the vertebrate central nervous system (Armstrong and Hawkes, 2000). Moreover, it is widely believed that the cerebellar cortex plays a coordinating role in memory, cognition, emotion, perception, and volitional movement (Buckner et al., 2011). In animal experiments, Garcia et al. (2012) found that exercise could cause intense changes in certain proteins, such as neurofilaments, in the cerebellar cortex of mice. In human experiments, Callow et al. (2021) also found that exercise training was associated with increased mean diffusivity in the cerebellum cortex. In our study, we found that physical exercise could effectively improve executive function in the elderly population, which was consistent with other research conclusions (Best et al., 2014; Xiong et al., 2021). But interestingly, we found that the right cerebellar cortex played a partial mediating role in the process of improving executive function through physical exercise. Therefore, we speculated that the cerebellar cortex may be more sensitive to physical exercise than the hippocampus and gray matter. As for why the left cerebellar cortex did not have similar performance, we guessed it might be related to the small sample size, or the left and right cerebellar cortex itself might have different functions.

We must admit that there are some limitations in our study. Firstly, information on physical exercise is obtained through self-report rather than objective assessment, so there is the possibility of recall bias. Secondly, since this study is only a cross-sectional study, it cannot indicate the causal effect between physical exercise and cognitive impairment. Thirdly, we don’t know if direct stimulation of the right cerebellar cortex can achieve the effect of physical exercise, therefore, a larger longitudinal study is needed to further confirm our findings.

## Conclusion

In conclusion, physical exercise among Chinese elderly is associated with a lower incidence of mild cognitive impairment and dementia, and there might be an association among physical exercise, executive function, and the thickness of the cerebellar cortex.

## Data availability statement

The raw data supporting the conclusions of this article will be made available by the authors, without undue reservation.

## Ethics statement

The studies involving human participants were reviewed and approved by the Ethics Committee of Shanghai Mental Health Center. The patients/participants provided their written informed consent to participate in this study.

## Author contributions

WL, YL, and LY contributed to the study concept and design. WL wrote this article. SX and YC analyzed the data and drafted the manuscript. All authors read and approved the final manuscript.
